# A scanning acoustic microscope discriminates cancer cells in fluid

**DOI:** 10.1038/srep15243

**Published:** 2015-10-19

**Authors:** Katsutoshi Miura, Seiji Yamamoto

**Affiliations:** 1 Department of Health Science, Pathology and Anatomy, Hamamatsu University School of Medicine, Hamamatsu, Japan; 2 Medical Photonics Research Center, Hamamatsu University School of Medicine, Hamamatsu, Japan

## Abstract

Scanning acoustic microscopy (SAM) discriminates lesions in sections by assessing the speed of sound (SOS) or attenuation of sound (AOS) through tissues within a few minutes without staining; however, its clinical use in cytological diagnosis is unknown. We applied a thin layer preparation method to observe benign and malignant effusions using SAM. Although SAM is inferior in detecting nuclear features than light microscopy, it can differentiate malignant from benign cells using the higher SOS and AOS values and large irregular cell clusters that are typical features of carcinomas. Moreover, each single malignant cell exhibits characteristic cytoplasmic features such as a large size, irregular borders and secretory or cytoskeletal content. By adjusting the observation range, malignant cells are differentiated from benign cells easily using SAM. Subtle changes in the functional and structural heterogeneity of tumour cells were pursuable with a different digital data of SAM. SAM can be a useful tool for screening malignant cells in effusions before light microscopic observation. Higher AOS values in malignant cells compared with those of benign cells support the feasibility of a novel sonodynamic therapy for malignant effusions.

Finding malignant cells and determining their cellular origin are critical for patient care. Effusion specimens from body cavities are usually diagnosed as benign, such as inflammation, or malignant, such as carcinoma, using a light microscope (LM). Because LM cytology requires special staining and a significant amount of labour for clinical diagnosis, an ancillary or screening method would be beneficial if available. Therefore, it would be interesting to assess whether a scanning acoustic microscope (SAM), which uses 100 MHz or higher frequency ultrasound, could help diagnose effusion cytology. SAM captures cellular images on a slide by plotting data related to the cell thickness, speed of sound (SOS) and attenuation of sound (AOS) through cells without staining (Fig. 1)^1^. Lemons and Quate^2^ first used SAM for cellular imaging in 1975. Since then, many researchers have used SAM to observe subcellular components. However, most samples analysed till date were cultured cells such as fibroblasts^3^, chicken heart cells^4^, HeLa cells^5^ and acute leukemic cells^6^, and SAM has not yet been used for the cytological diagnosis of real clinical samples. In the present study, we observed cytology specimens from patient body fluids using SAM to test the feasibility of its use. During LM observations, the nuclei and cytoplasm of the cell are stained for diagnosis, whereas SAM scans the cell without staining. SAM can provide cellular information regarding viscosity using AOS, elasticity using SOS^1^,^7^ and cellular thickness. In this study, we applied SAM to discriminate various free cells in fluid and compared the resulting images with those obtained using LM to assess the feasibility of SAM for cytological diagnosis.

## Results

### Characteristic cell images obtained using SAM

SAM could visualise not only SOS, AOS and the thickness of each cell but also whole cell sizes, borders, contents, cell connections and structures, similar to LM. Each cell type had its own mechanical properties that made characteristic acoustic images equivalent to optic images. Inflammatory cells and malignant lymphoma cells were usually present as a single cell, whereas epithelial cells including carcinomas and mesothelial cells often contained cell clusters. Generally, cell clusters exhibited larger SOS and AOS values compared with those of the surrounding single cells, which were detected easily using SAM. To compare the acoustic properties among each cell, all SOS, AOS and thickness ranges were standardised from 1485 to 1750 m/s, 0 to 9.0 dB/mm and 0 to 18.0 μm, respectively.

### Cell type-specific properties

#### Squamous cell carcinoma

Keratinizing squamous cell carcinoma (SCC) presented with a large polygonal shape and thick bumpy cytoplasm (Fig. 2A). The outer contours were irregular, and the cell size was 10 times larger than that of the inflammatory neutrophils observed in the background. The SOS and AOS values were the highest in the thickened cytoplasm.

#### Adenocarcinoma

Large adenocarcinomas (ADCs) of the pancreatic duct primary were present as single cells or multicellular clusters and were intermingled with small inflammatory cells (Fig. 2B). Individual ADCs had a large and irregular shape and displayed greater AOS, larger SOS and thicker cytoplasm than small inflammatory cells. Tumour cell clusters with large bulky masses exhibited a higher SOS and AOS than single tumour cells.

Breast ductal cell carcinomas in the pleural effusions formed ‘cannonballs’, which were very large balls of cells (Fig. 2C), as well as single cells. The outer contour of the cannonballs and the single cells was irregular. The SOS and AOS values were much higher than those of small inflammatory cells or erythrocytes.

Signet ring cell carcinoma of the stomach in the ascites was composed chiefly of single individual cells and some small groups of a few cells (Fig. 2D). Although the distribution of the cells was even, the size and shape of each cell varied. The SOS and AOS values were uneven among cells as well as within a cell. Specifically, the periphery of the cell had a higher AOS value than the centre.

#### Malignant mesothelioma

Malignant mesothelial cells in the pleural effusion were larger than inflammatory cells and often gathered to form grape-like groups (Fig. 2E) or were arranged in a line. Individual mesothelial cells had a round shape and were various sizes. The SOS and AOS values were greater than those of inflammatory cells. In addition, the SOS and AOS were lower in the centre of the cell than at the periphery.

#### Reactive mesothelial cells

Reactive mesothelial cells in inflammatory pleural effusion consisted of small round cells with a uniform size and shape (Fig. 2F). The mesothelial cells were slightly larger than the neutrophils and sometimes fused together to form small groups. The AOS and SOS values of reactive mesothelial cells were rather even and were higher than those of neutrophils but lower than those of mesotheliomas.

#### Malignant lymphoma

Malignant lymphoma cells were medium sized and slightly larger than inflammatory cells; they were present as single cells (Fig. 2G). The individual lymphoma cells exhibited lower SOS and AOS values compared with the other cancer cells.

#### Inflammatory cells

Inflammatory ascites or pleural effusions consisted of many neutrophils, lymphocytes and macrophages (Fig. 2H). The inflammatory cells presented as small round cells with a uniform size, shape and thickness. The SOS and AOS values were lower than those of the neoplastic cells. It was difficult to obtain acoustic images from cells that were too small in size or were too thin.

### Statistical analysis

The means and standard deviations (SDs) of AOS, SOS and cell thickness for SCC, ADC, mesotheliomas, reactive mesothelial cells, lymphomas and inflammatory cells are summarised in Fig. 3 and Table 1. Each cell type exhibited characteristic SOS values (Fig. 3A), AOS values (Fig. 3B) and thickness (Fig. 3C), and there were statistically significant differences among them. Generally, carcinomas had greater SOS, AOS and thickness than inflammatory cells and lymphomas. Larger cells had a tendency to possess a greater SOS and AOS.

When malignant and benign effusions were compared, the former has higher SOS and AOS values than the latter, whereas there was no significant difference in the mean thickness between the two cell types. The sensitivity and specificity of cytological examinations by SAM and LM were compared in 145 recent cases of effusion cytology. The specified cutoff SOS and AOS values to differentiate malignant and benign cases were 1628.0 m/s and 4.29 dB/mm respectively. The sensitivity and specificity for SOS were 91.7% and 91.8%, respectively, and those for AOS were 86.7% and 74.1%, respectively. Under these conditions, when > 10% single cells or small clusters showed values higher than the cutoff values, they were regarded as malignant cases by SAM (Table 2). By adjusting the observation range, malignant cells could be differentiated from benign cells easily using SAM.

When epithelial cells and white blood cells were compared, epithelial cells had higher SOS, larger AOS and greater thickness values than white blood cells.

Among the different types of ADC, the values of AOS differed depending on the cytoplasmic content. Signet ring cell carcinoma, which contained significant amounts of mucin in the cytoplasm, had a higher AOS than other serous carcinomas or lung ADCs (Fig. 4, Table 3). The values of AOS had a significant difference between signet ring cell carcinoma and lung adenocarcinoma.

### Observational properties of SAM and LM

Table 4 summarises the advantages and disadvantages of SAM and LM as cytology methods. LM was superior for observing nuclear characteristics, such as nucleus size and chromatin density, whereas SAM provided superior resolution of cytoplasmic characteristics such as cell size, border, content and irregularity. The observation range is adjustable using SAM; therefore, discriminating between malignant and benign cells is easier than with LM. Statistical analysis is also easier and faster using SAM compared with LM.

## Discussion

The acoustic properties of different cell types were distinct enough to make characteristic acoustic images. We recently imaged various tissues using SAM, including the lungs^8^, stomach^9^, thyroid^10^ and lymph nodes^11^. Statistically significant differences in SOS, AOS and the thickness of each cell may help discriminate cell types. For cellular imaging, previous studies obtained the mechanical properties of cells from living skin fibroblasts^12^ and a HeLa cell^13^, although the resolution of these images was poorer than that achieved in the current study. Many mechanical functions of the cell are derived from and controlled by the cytoskeleton, which serves as an intracellular scaffold to supports the cell’s shape, movement and force^14^,^15^. The cytoskeleton consists of three distinct types of polymer biomolecules: actin microfilaments, intermediate filaments and microtubules^16^. Among the different cytoskeletal filaments, F-actin provides the highest resistance to deformation until a certain critical local strain^15^. Actin forms networks at the cell cortex, where they can easily ‘fluidize’ under high shear stresses to facilitate cell locomotion. Intermediate filaments are sufficiently compliant to generate moderate deformation and yet maintain resistance to shear deformation in response to large local strains to provide structural integrity to the cell. In contrast, microtubules do not have sufficient tensile or shear stiffness to impart significant mechanical integrity to the cytoskeleton.

F-actin is prominent in microvilli, which are finger-like protrusions of the plasma membrane found in cells involved in absorption and secretion. For example, mesothelial cells have numerous long villi^17^. Mesothelioma cells exhibited a lower SOS and AOS at the periphery compared with the centre of the cell in the current study.

Intermediate filaments constitute approximately 1% of the total proteins in most cells but can account for up to 85% in cells such as epidermal keratinocytes and neurons^15^. Keratin and vimentin intermediate filaments predominate in epithelial and non-epithelial cells, respectively. Generally, malignant epithelial tumours such as carcinomas have keratin intermediate filaments, whereas malignant non-epithelial tumours such as sarcomas possess vimentin filaments^18^. There are at least 30 different types of keratin proteins. The attachment of keratins to the epithelial sheet provides it with mechanical rigidity and cell–cell adhesion junctions known as desmosomes. Generally, carcinomas are harder than sarcomas by experience. The current study supported the hardness of carcinoma by comparing SOS data among carcinomas and lymphomas because harder tissues exhibited greater SOS.

Microtubules are important for cell migration, particularly during mitosis. Poorly differentiated tumours exhibit more mitotic activity than well-differentiated tumours. Our previous report^10^ showed that a poorly differentiated carcinoma has a lower SOS value than a well-differentiated carcinoma, which means that the former is softer than the latter.

As a general rule of cytological diagnosis, malignant cells are larger and more pleomorphic than normal or reactive cells, and they have increased nuclear to cytoplasmic ratios. Abnormal, coarse, dark and irregularly distributed chromatin are key diagnostic features of malignant cells^19^. Although detailed nuclear features cannot be visualised using SAM, large pleomorphic cytoplasm in malignant cells can be visualised using this technique. Epithelial cell carcinomas have a tendency to form clusters, which can be observed easily using SAM because of their large size and high SOS and AOS values.

In the current study, SAM could detect each characteristic feature of malignant cell types. SCC was characterised by a thick dense cytoplasm with distinct borders. In addition, SAM could visualise diagnostic features such as irregular shapes and thick keratinisation in the cytoplasm. Keratinizing SCC exhibited the highest SOS and AOS values among all carcinomas.

ADCs exhibited different patterns on SAM depending on the cellular origin. Several typical SAM patterns were observed in the current study. Papillary structures, which are three-dimensional clusters that are longer in one direction than in the other two, are suggestive of gastrointestinal tract or ovarian cancer in ascetic fluids, and lung, breast cancer or mesothelioma in pleural fluids^19^. Signet ring cells are suggestive of gastric or breast carcinoma. Intracytoplasmic mucin had a higher AOS value, whereas cannonballs suggested that the cells were breast carcinomas. These SAM observations suggest that the ADC cell type could be identified using these typical patterns and acoustic values.

The most characteristic feature of malignant lymphomas is that all cells are single, and there are no true tissue aggregates. SAM could display the characteristic single cell features successfully. Regarding malignant mesothelioma, clusters of cells with irregular, knobby, flower-like outlines are characteristic^19^, whereas ADC clusters usually have smooth borders. Numerous cell aggregates and long chains of cells are more common in mesothelioma compared with benign effusions. Individual malignant mesothelial cells are usually larger and more variable in size and shape than are benign mesothelial cells. The acoustic SAM images obtained in the current study reflected these typical features of mesothelioma. When the AOS and SOS values of malignant tumours and benign cells were compared, significant differences were observed (*P* < 0.01), although there was no significant difference in cell thickness.

A novel sonodynamic therapy (SDT) has been established and developed as a cancer treatment using similar principles to photodynamic therapy^20^. SDT is an ultrasound-based approach that involves the use of low-intensity ultrasound and a chemical (a sonosensitizer or a chemotherapeutic drug) that can be activated by sonication. Therapeutic ultrasound also uses direct thermal effects^21^. Cancer cells can be damaged by heat or cytotoxic drugs more readily than can normal cells. Unlike visible light, ultrasound waves can penetrate a cancer target buried deep within human body. Therefore, SDT is a feasible treatment for malignant effusions. Malignant cells have a tendency to exhibit a high AOS; therefore, malignant cells are more susceptible to ultrasound thermal damage than normal cells. This difference in acoustic properties might be available for exploitation in cancer treatment, particularly in tumours with high AOS values. AOS correlates with the viscosity^22^, and so mucin-containing cancers such as signet ring cell carcinomas are appropriate targets.

SAM has five unique features compared with optical microscopy. First, the measurement procedure is rapid and easy, without a requirement for special staining. Therefore, SAM is suitable for the initial screening of cells, and intact cells are available for further staining or genome analysis. Second, the observation ranges of SOS, AOS and thickness can be adjusted, which facilitates the detection of specific cells such as malignant cells. Most malignant cells were discriminated by high SOS or AOS values. Third, observations are repeatable and can be compared under different conditions and cells, such as before and after chemical modification^23^ or therapy. Fourth, the microscopic acoustic properties correspond to the echo intensity and texture in clinical echography. Finally, SAM data can help assess the biomechanical characteristics of tissues and cells, such as elasticity and viscosity^1^.

There are three limitations to the current SAM method. First, the thickness of cells needs to be > 3 μm to calculate SOS and AOS. Second, high background contamination can interfere with correct calculations. For example, mineral crystals, artificial fibres and cell debris all interfere with correct measurements. Third, fixation methods affect SOS, AOS and thickness measurements. In the current study we used 95% ethanol as a fixative. Our preliminary data suggest that the addition of formalin increases the SOS and AOS values and decreases thickness^23^.

In the current study, we applied the thin layer preparation method to the effusion cells. This method can also be applied to solid tumours by puncturing the tumours with fine needles or scrubbing the tumour surface and then fixing in ethanol solution.

In conclusion, cytological diagnosis using SAM without additional staining is of great value for differentiating between benign and malignant effusions. Although SAM is inferior to LM for detecting nuclear features, it can differentiate malignant from benign cells using SOS and AOS. Moreover, each malignant cell exhibited characteristic cytoplasmic features such as a large size, irregular borders and distinctive content. Large cell clusters with irregular outlines were also typical features of carcinomas. SAM will be a useful tool for screening malignant cells in effusions. In addition, the high AOS values in malignant cells suggest that SDT is a feasible treatment option. Subtle changes after treatment and the functional heterogeneity of tumour cells could be observed using SAM.

## Methods

### Study Design

**Figure 1:**
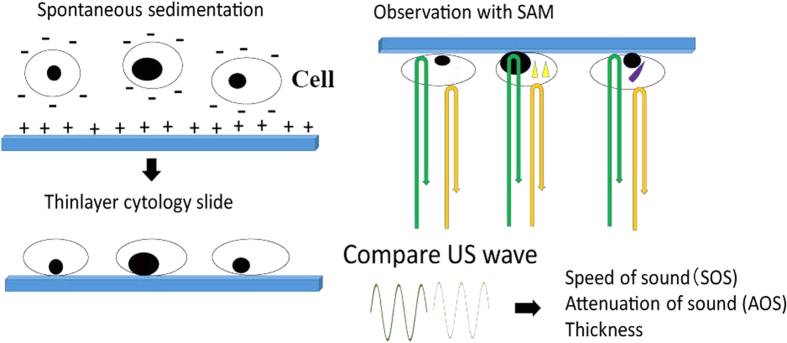
Study design. Free cells of effusion were fixed in 95% ethanol and centrifuged to make precipitates. Then, the precipitates were washed in distilled water, centrifuged again and poured on a glass slide. The negatively charged cells spontaneously settled on a positively charged slide to form a thin-layer specimen. The cytologic specimen was scanned with US probe to compare the US waves from the surface of the cell and glass slide. The speed of sound (SOS) and the attenuation of sound (AOS) through cells and the thickness of cell were calculated to generate images on screen.

**Figure 2:**
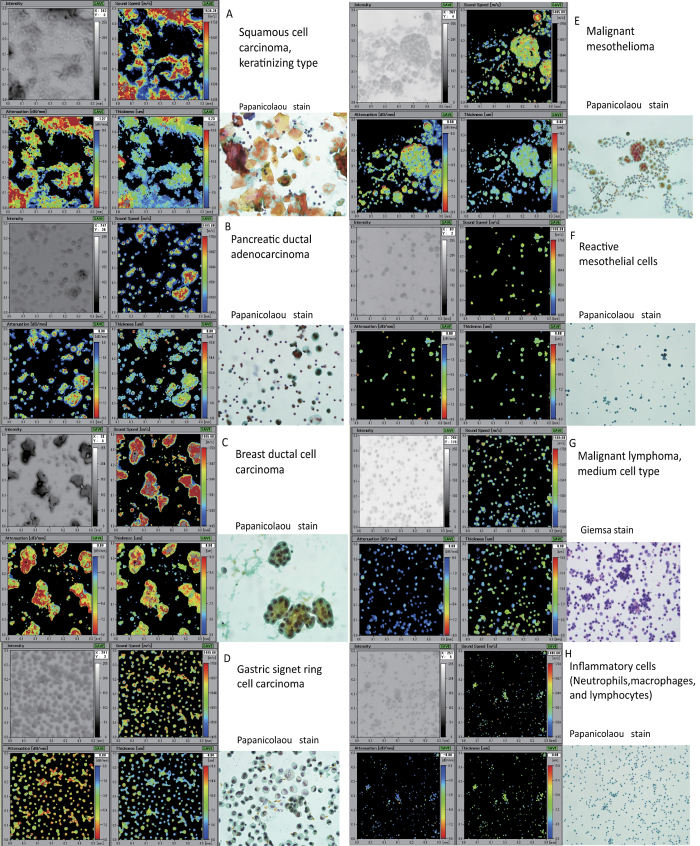
Images of various cells obtained using a scanning acoustic microscope (SAM). Images of acoustic intensity (upper left), speed of sound (SOS; upper middle), attenuation of sound (AOS; lower left) and thickness (lower, middle) are shown. Light microscope (LM) slides from the same cytology samples were prepared using Papanicolaou or Giemsa stain (lower right). (**A**) Keratinizing squamous cell carcinoma (SCC) exhibited a large polygonal shape with irregular contours and thick bumpy cytoplasm. The SOS and AOS values were the highest in the thickened cytoplasm. (**B**) Adenocarcinoma (ADC) cells of the pancreas were in the single celled or multicellular cluster form and were intermingled with small inflammatory cells. Individual ADCs had a large and irregular shape and exhibited a higher AOS, larger SOS and thicker cytoplasm than inflammatory cells. The tumour cell clusters with large bulky masses had higher SOS and AOS than single tumour cells. (**C**) Breast ductal cell carcinomas formed cannonballs, which were very large balls of cells, as well as single cells. The SOS and AOS were much higher than those of small inflammatory cells. (**D**) A gastric signet ring cell carcinoma was composed mainly of single individual cells. Although the distribution of the cells was even, the size and shape of each cell varied. The SOS and AOS values were uneven both among cells and within individual cells. (**E**) Malignant mesothelial cells were larger than inflammatory cells and often gathered to form grape-like groups or were arranged in a line. Individual mesothelial cells had a round shape and were various sizes. The SOS and AOS values were greater than those of inflammatory cells. (**F**) Reactive mesothelial cells consisted of small round cells of a uniform size and shape. The AOS and SOS values of reactive mesothelial cells were higher than those of neutrophils. (**G**) Malignant lymphoma cells were a little larger than inflammatory cells and presented as single cells. The lymphoma cells exhibited lower SOS and AOS values compared with other cancer cells. (**H**) Inflammatory pleural effusions consisted of many neutrophils, lymphocytes and macrophages. The cells presented as small round cells of a uniform size, shape and thickness.

**Figure 3:**
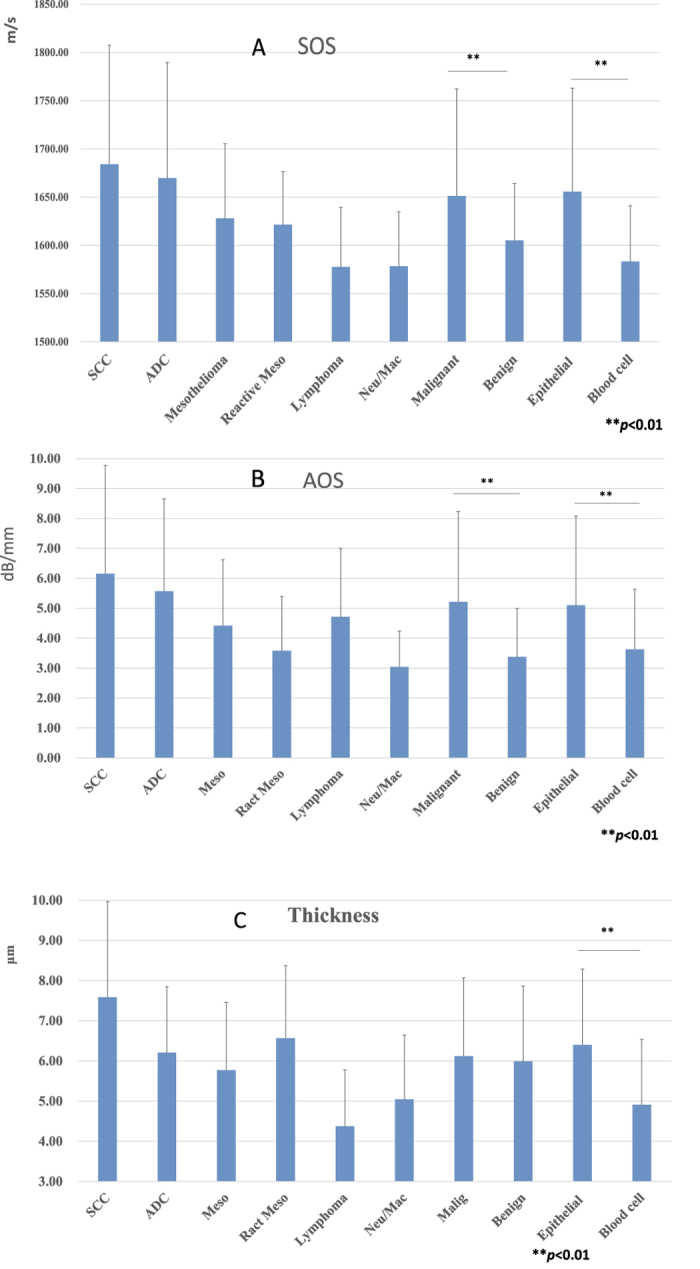
Acoustic values of speed of sound (SOS), attenuation of sound (AOS) and thickness. (**A**) There were significant differences in SOS among cell types. The malignant cell group had significantly greater SOS values than those of the benign cell group, whereas the epithelial group had higher SOS values than the blood cell group (*P* < 0.01). (**B**) There were significant differences in AOS among cell types. The AOS values of the malignant and epithelial cell groups were significantly greater than those of the benign and blood cell groups, respectively (*P* < 0.01). (**C**) The cell thicknesses differed among each cell type. Generally, thicker cells had a higher SOS and AOS. The epithelial cell group was significantly thicker than the blood group, but there was no significant difference between the malignant and benign groups (*P* < 0.01). SCC, squamous cell carcinoma; ADC, adenocarcinoma; Meso, mesothelioma; React Meso, reactive mesothel; Neu/Mac, neutrophil/macrophage.

**Figure 4:**
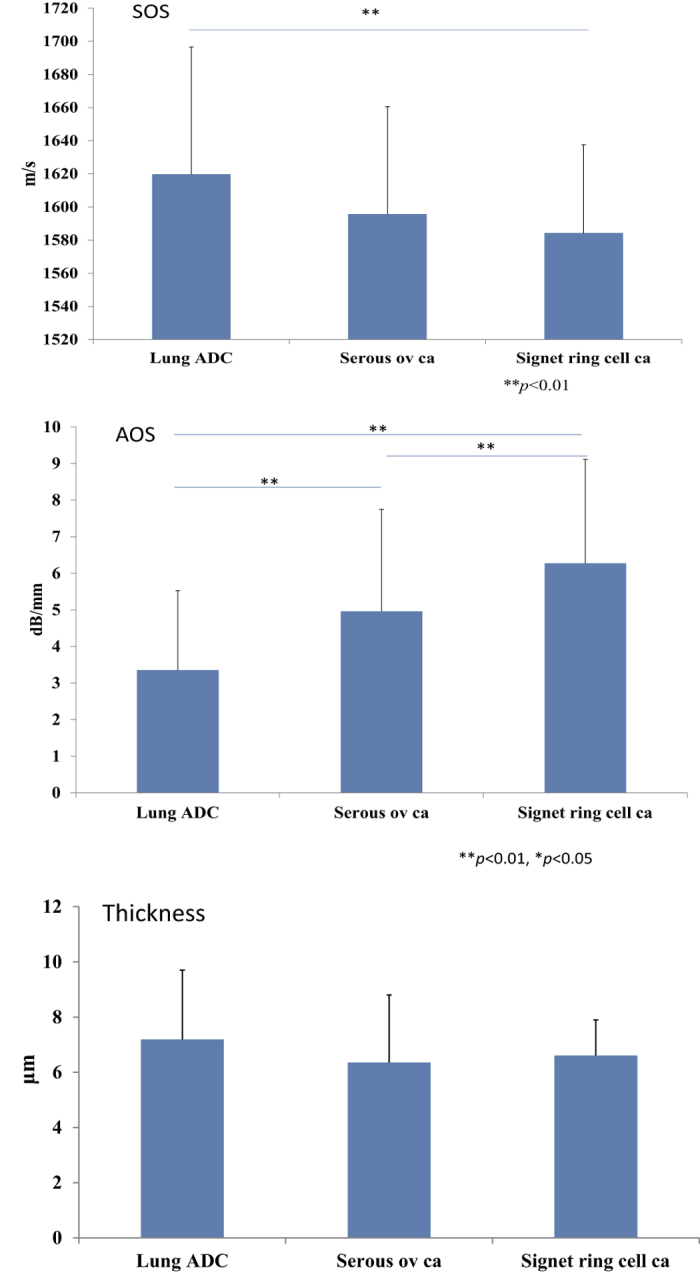
SOS, AOS and thickness variation among different ADCs. Signet ring cell carcinomas, which contained large amounts of mucin in the cytoplasm, had a higher AOS than that of other serous carcinomas or lung ADCs. SOS values showed significant difference between signet ring cell carcinomas and ADCs of the lung. There were no significant differences among each thickness value.

The protocol for using residual cytology samples without a link for patient identity was approved by the Ethics Committee of Hamamatsu University School of Medicine. Informed consent was obtained from all subjects. Residual free cells from ascites, pleural effusions, pericardial effusions and cystic fluids were prepared to make single cell-layer slides using a liquid-based cytology method (BD CytoRich™; Franklin Lakes, NJ, USA). This method was used to collect cells on the slide by ionic bind. The cells were negatively charged and spontaneously precipitated on the slide which was positively charged (Fig. 1). The slides were fixed in 95% ethanol or BD CytoRich™ preservative and kept dry until observation. The slide was then re-soaked in distilled water and observed using SAM. The specimens consisted of SCCs, ADCs, reactive mesothelial cells, mesotheliomas, malignant lymphomas, neutrophils, macrophages and lymphocytes. Neutrophils, macrophages and lymphocytes were classed together as inflammatory cells because SAM could not discriminate among these small inflammatory cells easily. At least three different cases were selected from each cell type, and at least three different areas of each case were observed using SAM. Cases with severe degenerative changes after chemotherapy or radiation therapy were excluded. All cases were diagnosed using routine cytology or histology. The methods were carried out in accordance with the approved guidelines.

### Observation by SAM

The SAM (AMS-50AI) was supplied by Honda Electronics (Toyohashi, Aichi, Japan) and was equipped with a 400-MHz transducer with a resolution of 3.75 μm, which is capable of cellular imaging. The SAM used a single pulsed wave to image an object^24^. Each specimen was scanned in a frame with 300 × 300 pixels, and an area 300 × 300 μm^2^ was scanned to obtain each image within 3 min. The two-dimensional distribution of the echo intensity of sound, SOS and AOS through the cells, as well as the cellular thickness was obtained and plotted^8^. The penetration depth was up to a maximum of 20 μm. The observation range within which the values were plotted could be adjusted.

### Statistical analysis

The acoustic data from 64 cross points on the lattice screen of each image were collected and statistically analysed. SOS, AOS and thickness of individual cells were counted. Cell clusters with a thickness >15 μm were excluded. The data from at least three different cases were unified into means ± SDs for each cell type. For comparisons between malignant and benign cells, SCCs, ADCs, mesotheliomas and malignant lymphomas were included in the malignant group, whereas reactive mesothelial cells and inflammatory cells were considered benign. For comparisons between epithelial cells and white blood cells, SCCs, ADCs, mesotheliomas and reactive mesothelial cells were included in the epithelial group, whereas malignant lymphomas and inflammatory cells formed the blood cell group. Student’s *t*-tests were used for determining statistical differences among specimens, and *P* < 0.05 was considered statistically significant.

### Light microscopic observation

To compare SAM and LM images, slides from the same cytology samples were prepared using Papanicolaou stain or Giemsa stain.

## Additional Information

**How to cite this article**: Miura, K. and Yamamoto, S. A scanning acoustic microscope discriminates cancer cells in fluid. *Sci. Rep*. **5**, 15243; doi: 10.1038/srep15243 (2015).

## Figures and Tables

**Table 1:** Acoustic SOS and AOS values and the thickness of various cells.

SOS			
	n	Average (m/s)	SD
SCC	111	1684.10	123.46
ADC	356	1669.61	120.10
Mesothelioma	132	1627.94	77.47
Reactive Meso	130	1621.50	54.80
Lymphoma	85	1577.56	61.55
Neutro/MΦ	81	1578.38	56.23
Malignant	711	1651.10	110.99
Benign	211	1604.95	59.09
Epithelial cell	729	1655.69	107.31
Blood cell	193	1583.30	57.85
AOS			
	n	Average (dB/mm)	SD
SCC	110	6.15	3.62
ADC	365	5.57	3.09
Mesothelioma	131	4.42	2.20
Reactive Meso	130	3.58	1.82
Lymphoma	85	4.71	2.29
Neutro/MΦ	81	3.04	1.19
Malignant	718	5.21	3.02
Benign	211	3.37	1.62
Epithelial cells	736	5.10	2.98
Blood cell	193	3.63	2.00
Thickness			
	n	Average (μm)	SD
SCC	110	7.58	2.38
ADC	363	6.21	1.64
Mesothelioma	131	5.77	1.68
Reactive Meso	130	6.57	1.80
Lymphoma	85	4.38	1.40
Neutro/MΦ	79	5.04	1.60
Malignant	716	6.12	1.94
Benign	209	5.99	1.88
Epithelial	734	6.40	1.88
Blood cell	191	4.91	1.63

ADC, adenocarcinoma; AOS, attenuation of sound; Neutro/MФ, neutrophils/macrophages; Reactive Meso, reactive mesothelial cells; SCC, squamous cell carcinoma; SD, standard deviation; SOS, speed of sound.

**Table 2:** Comparison of LM and SAM to differentiate malignant and benign cases.

SOS of SAM*	LM malignant	Benign
Malignant	55	7
Benign	5	78
AOS of SAM*	LM malignant	Benign
Malignant	52	22
Benign	8	63

^*^Cutoff points for SOS and AOS values to differentiate malignant and benign cells were set at 1628.0 m/s and 4.29 dB/mm, respectively. Malignant epithelial cells and benign cells were differentiated by SOS or AOS values only.

**Table 3:** SOS, AOS and thickness variation among different ADCs.

SOS			
	n	Ave (m/s)	SD
Lung ADC	110	1619.74	76.86
Serous ov ca	38	1595.60	65.02
Signet ring cell ca	108	1584.19	53.35
AOS			
	n	Ave (dB/mm)	SD
Lung ADC	166	3.35	2.17
Serous ov ca	39	4.96	2.78
Signet ring cell ca	109	6.27	2.84
Thickness			
	n	Ave (um)	SD
Lung ADC	165	7.19	2.51
Serous ov ca	39	6.35	2.45
Signet ring cell ca	108	6.61	1.29

**Table 4:** Comparative features of SAM and LM cytology.

Cytological analysis		SAM	LM
Nucleus	Size	△	⊚
	Irregularity/groove	X	⊚
	Chromatin density	X	⊚
Cytoplasm	Size	⊚	○
	Border	⊚	○
	Content	⊚	○
	Irregularity	⊚	△
	Thickness	⊚	△
Cell connection		⊚	⊚
Group structure		⊚	⊚
Observation range	adjustability	⊚	X
Statistical analysis		⊚	○

SAM, scanning acoustic microscope; LM, light microscope; ⊚, excellent; ○, good; △, difficult; X, impossible.
